# Immune Reconstitution Inflammatory Syndrome and Cytomegalovirus Pneumonia Case Report: Highlights and Missing Links in Classification Criteria and Standardized Treatment

**DOI:** 10.1155/2017/9314580

**Published:** 2017-09-18

**Authors:** Stefania Petarra-Del Río, Adrian Rodriguez-Hernandez, Luis Anguiano-Landa, Georgina Aguilar-Portillo, Isidro Zavala-Trujillo, Arnulfo Hernan Nava-Zavala, Maria G. Zavala-Cerna

**Affiliations:** ^1^Immunology Research Laboratory, International Program of Medicine, Universidad Autonoma de Guadalajara, Zapopan, JAL, Mexico; ^2^Infectious Disease Department, Hospital Dr. Angel Leaño, Universidad Autonoma de Guadalajara, Zapopan, JAL, Mexico; ^3^Pathology Department, International Program of Medicine, Universidad Autonoma de Guadalajara, Zapopan, JAL, Mexico; ^4^UIEC, Hospital de Especialidades del Centro Médico Nacional de Occidente, Instituto Mexicano del Seguro Social, Guadalajara, JAL, Mexico

## Abstract

**Background:**

Cytomegalovirus (CMV) pulmonary involvement is rarely associated with IRIS; therefore, limited information is available.

**Case Presentation:**

Here, we describe the case of a 43-year-old HIV-infected male who developed an unusual case of IRIS after cytomegalovirus (CMV) pneumonia. Clinically there was a progressive and paradoxical worsening of respiratory distress, despite being treated for CMV after initiation with antiretroviral therapy. Chest X-ray revealed disseminated infiltrates in both lungs; chest CT-scan showed generalized lung involvement and mediastinal adenopathy. Pulmonary biopsy confirmed CMV pneumonia with the observation of typical viral inclusions on pneumocytes.

**Conclusions:**

CMV pneumonia can be associated with the development of IRIS requiring treatment with immunosuppressant's and immunomodulatory drugs.

## 1. Introduction

During the acute phase of human immunodeficiency virus (HIV) infection, patients are typically asymptomatic; activation of the immune system is crucial but eventually replication of the virus inside CD4+ T-cells induces a cytopathic effect responsible for subsequent deterioration of immune responses, rendering acquired immunodeficiency syndrome (AIDS) patients at risk of developing opportunistic infections (OI). Cytomegalovirus (CMV) can cause OI in AIDS patients, which usually induces robust CD8 + and CD4+ T-cell-mediated immune responses associated with the resolution of the acute primary infection; then after immunosuppression (CD4+ T-cell count < 50 cells/*µ*L) the virus establishes reactivation and persistent infection with hematogenous spread [1]. CMV is a member of the Beta Human Herpesvirus class, with a double stranded DNA virus of approximately 220 kb, easily transmitted by contact with bodily fluids. CMV seroprevalence varies per country, socioeconomic class, and geographic locations; overall, in adult population it ranges from 40 to 100% [2, 3]. Primary infection in healthy individuals is usually asymptomatic, although approximately 10% develop mononucleosis-like syndromes [2]. The spectrum of CMV disease is wide, including congenital infections, disease in transplant recipients, and ocular and esophageal diseases in HIV-patients. Additionally, in HIV-patients with a CD4+ T-cell count < 100, CMV is a common cause of retinitis [3, 4]. ART has proven to be effective and it is recommended that patients with HIV infection and detectable viremia begin ART regardless of their CD4+ cell count [5]. Furthermore, when patients have an acute OI, ART should begin no later than 2 weeks; however, one important consideration upon ART initiation is the possibility of developing immune reconstitution inflammatory syndrome (IRIS), a potentially severe complication that reflects an overreaction of the pathogen-specific immunity, due to immune reconstitution [6]. IRIS is thought to occur after recovery of the adaptive immune system, leading to a hyperinflammatory response to a previously acquired OI. It is often the result of CD4+ T-cells proliferation but paradoxically patients have a worse clinical outcome due to excessive inflammatory responses to a previous evident or masked underlying OI, despite treatment [7]. This inflammatory response could be against viable or nonviable residual antigens from different organisms [8]. IRIS can be observed consequently after a wide variety of pathogens with different clinical presentations; however, it is generally characterized by persistent fever and significant clinical deterioration, with systemic and local involvement depending on the pathogens involved [9]. IRIS affects approximately 10–32% of HIV-patients. Variations in the frequency reflect the difference in case definitions, study populations, risk factors, and OI. Furthermore, IRIS can be classified into two different types, paradoxical IRIS and unmasking IRIS [8]. Paradoxical IRIS entails a previously detected OI that worsens after ART initiation. Conversely, unmasking IRIS occurs in a patient with subclinical OI that worsens with recovered immune response after ART. Both forms of the disease lead to inflammation and tissue destruction with clinical and radiological evidence and are associated with increased mortality rate [10, 11]. Moreover, 37.7% of patients with a diagnosis of CMV-retinitis prior to antiretroviral therapy (ART) developed IRIS, compared to 6.4% of patients with Kaposi sarcoma (KS) [5]. CMV pneumonia is an important cause of morbidity and mortality in immunocompromised patients, but it is a rare form of presentation and has not been linked to the development of IRIS previously [12]. Here we present a patient with pulmonary involvement due to CMV, who presented severe deterioration after ART onset and raised the possibility of suffering from IRIS. We provide information on the establishment of IRIS and offer considerations required for effective treatment.

## 2. Case Presentation

**Figure 1:**
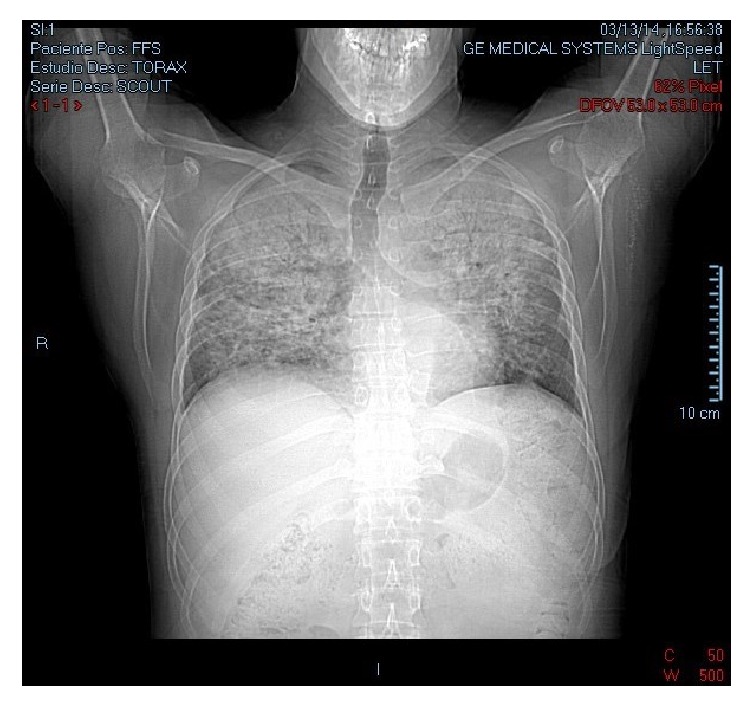
Pulmonary CT-scan with evidence of atypical bilateral pneumonia: pulmonary parenchyma with multiple areas of interstitial infiltrates (patchy infiltrates) involving apical and medial areas of both pulmonary fields.

A 43-year-old male patient presented to the emergency department with a 20-day history of fever, predominantly at nights and headaches. His previous medical history is unremarkable; he only referred to a 20-year history of smoking and denied use of intravenous or recreational drugs, any prior sexually transmitted diseases, or blood transfusions. At presentation, the patient had a blood pressure of 109/75 mmHg, heart rate 87 bpm, respiration rate 24 breaths/minute, temperature 38.5°C, and oxygen saturation of 70% at atmospheric pressure. Physical examination revealed pale skin and mucosa; tongue and soft palate had lesions consistent with oral candidiasis and congestive pharynx. Pulmonary fields revealed decreased sounds without crackles or wheezing and painful hepatomegaly and extremities with hyperchromic nodular lesions on both ankles, suggestive of Kaposi sarcoma. Due to these findings, initial blood tests included HIV serology that came out positive; CBC: Hb 11.1 g/dL, hematocrit 33.8%, WBC 7,100/mm^3^, lymphocytes 3%, and neutrophils 95%; IgE 788.2 UI/ml; VSG 77 mm/hr; TGO 91 UI/L; TGP 66 UI/L; and DHL 2250 UI/L. Chest X-ray revealed disseminated infiltrates in both lungs. Medical management was initiated with omeprazole, metamizole, and oxygen with nasal prongs 3 L/min. On day 2 of hospitalization, the bronchoscopy fluid examination resulted positive for* C. albicans* and negative for other pathogenic bacteria and fungi. However, we decided to start dexamethasone 6 mg IV, trimethoprim/sulfamethoxazole 160/800 mg, nebulization with ipratropium, and budesonide due to high suspicion of* P. jirovecii *infection. On day 3 of hospitalization, HIV infection was confirmed with a viral load of 531,000 copies/ml and CD4+ T-cell count of 11 cells/mm^3^. Other studies were performed including a PPD (negative test 0 mm) and anticytomegalovirus serology (IgG positive). On day 4 of hospitalization, the infectious disease division started ART with ritonavir/lopinavir, tenofovir, and emtricitabine. During the subsequent days, the patient showed clinical improvement. However, on day 13, his clinical condition declined with progressive dyspnea, severe dysphagia, and abdominal pain. Auscultation showed basal rales in both lungs; a new chest X-ray demonstrated no changes compared with the previous one. New blood tests included CBC: Hb 14.5 g/dl, hematocrit 44%, VCM 90 fL, platelets 284,000 × 10^3^, WBC 18,400/mm^3^, neutrophils 89%, and lymphocytes 5%. Serum electrolytes, glucose, BUN, and creatinine had no alterations. He developed dysphagia and episodes of oxygen desaturation partially corrected with nasal prongs. On day 19 of hospitalization, due to continuous episodes of low oxygen saturation, a chest CT-scan was ordered (Figure 1), showing generalized lung involvement and mediastinal adenopathy (Video 1 in Supplementary Material https://doi.org/10.1155/2017/9314580).

Supplementary Video 1 showed digitalized CT-scan of the thorax in coronal sectioning.

**Figure 2:**
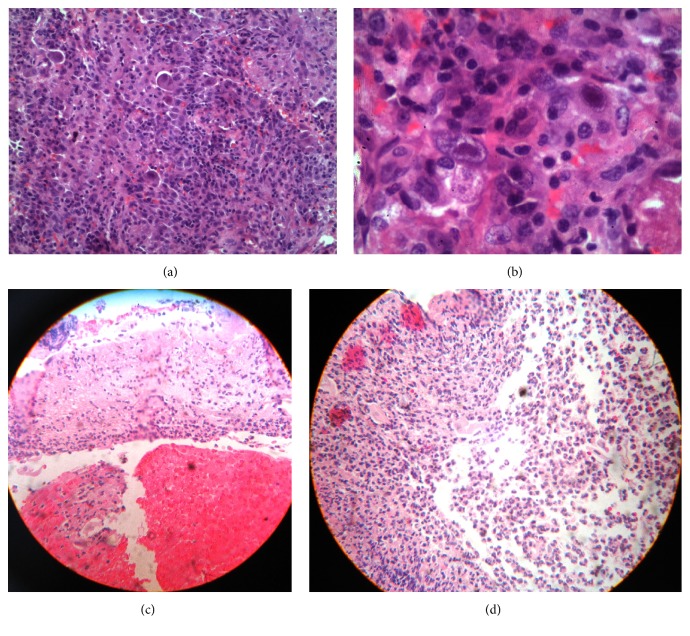
Panel (a): lung biopsy with pulmonary tissue showing young fibroblast proliferation at the interstitial level with lymphocytes, histiocytes, and red blood cells. Panel (b): abundant cytomegalovirus inclusions and alveolar spaces with type II pneumocytes and red blood cells. Ziehl-Neelsen, Grocott, and periodic acid-Schiff stain (PAS) were negative. Panel (c): esophagus biopsy with loss of mucosal continuity and the presence of hemorrhage. Panel (d): important infiltration of polymorphonuclear cells and epithelioid cells with a few fragments of squamous epithelia. Acid fast bacilli stain and fungal stains were negative.

**Figure 3:**
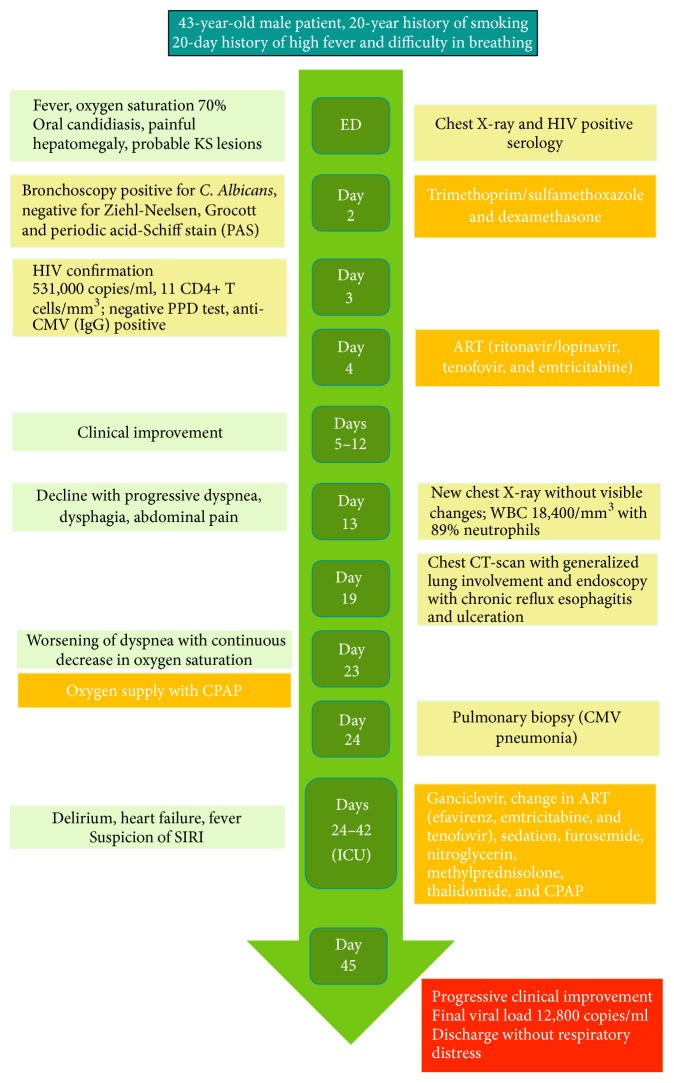
Timeline of patients' clinical evolution with diagnostic tests and treatments. HIV human immunodeficiency virus, KS Kaposi's sarcoma, PPD purified protein derivative, CMV cytomegalovirus, ART antiretroviral therapy, CPAP continuous positive airway pressure, and IRIS immune reconstitution inflammatory syndrome.

Later that day, an endoscopy showed chronic reflux esophagitis (Grade D of the Los Angeles Classification System), with ulceration (Figures 2(c) and 2(d)). On day 23 of hospitalization, dyspnea worsened; oxygen supply was administered with continuous positive airway pressure (CPAP). A pulmonary biopsy was performed on day 24, which reported CMV pneumonia (Figures 2(a) and 2(b)); the patient was transferred to the intensive care unit (ICU) due to persistent hypoxemia and was started on methylprednisolone 500 mg IV (maintained for 2 days) and ganciclovir 500 mg IV, and ART was changed to efavirenz, emtricitabine, and tenofovir, to decrease pill intake (1 pill/day). Intravenous sedation was initiated and oxygen supply was maintained with CPAP. The patient had 2 episodes of heart failure that required management with furosemide and nitroglycerin. He developed fever and respiratory distress with episodes of delirium treated with antipsychotics; we suspected the development of IRIS due to a paradoxical worsening of his condition despite being treated with ART and ganciclovir. Therefore, we started methylprednisolone and thalidomide 100 mg/day for immunosuppression and immunomodulation, respectively. After 17 days in the ICU, the oxygen requirements began to drop and the mental status improved. He remained hemodynamically stable with clinical and radiological improvemt. A new viral load reported 12,800 copies/ml. A new CBC reported hemoglobin 8 g/dL, hematocrit 26.4%, MCV 29 pg., platelets 366,000, WBC 7,400 mm^3^, neutrophils 86%, and lymphocytes 6%. Patient was discharged from the hospital after 45 days of treatment (Figure 3).

## 3. Discussion

Pathogenesis of IRIS has partially been described as a restoration in CD4+ T-cells following ART, allowing a rapid response of previously pathogen-specific innate immune responses with the infective organism determining the following overwhelming immunopathology. IRIS can present as soon as one week after the initiation of ART and up to 4 years; however, it commonly presents during the first 3 months [8]. Our case report describes an HIV patient that developed a severe inflammatory response, 19 days after the initiation of ART with severe pulmonary involvement. Since serum specific biomarkers are not yet available for IRIS, the diagnosis must be primarily clinical. In 2004, French et al. proposed classification criteria for IRIS diagnosis [13] (Table 1).

The present case report fulfilled one major criterion (A), 2 minor criteria (2 and 3), since CD4 + T-cell count was not performed afterwards, and no exclusion criteria. In this case or in other cases of SIRI, a possible association between numbers and phenotypes of CD4+ T-cells and clinical parameters should be established; this would provide important information about the pathogeny and recovery after IRIS. French et al. classification criteria for IRIS should be reviewed and additional parameters should be included such as time for the initiation of ART (1 week to 1 year) and perhaps more importantly other laboratory tests that reflect acute inflammatory responses should be added (CRP or ESR) [8]. Additionally, inflammatory cytokines could also be evaluated such as TNF and IL-1.

Previous attributable risk factors for IRIS development include CD4+ T-cell count below 50 cells/mL and a short interval between the initiation of ART and treatment for OI [9]; our patient fulfilled both. Since CD4+ T-cell count is the single most important risk factor, some authors have proposed that ART should be initiated with levels of CD4+ T-cell ≥ 350/*µ*L and at the same time tests should be performed to identify OI, while others suggest postponing ART for 4–8 weeks if an OI is clearly present, being conscious of the additional risk that carries the delay of starting this therapy [14].

IRIS incidence varies between developed and developing nations, with higher prevalence in the latter, which might be due to a delay in ART initiation; a previous study suggested that some individuals are genetically susceptible to develop IRIS with an exacerbated immune recovery due to a functional defect in regulatory T-cells [9]. Others state that HLA genes might render individuals susceptible to developing IRIS when determined pathogens are present as seen in herpes virus-IRIS and mycobacterial-IRIS or CMV-IRIS which frequently carries HLA-B44 and ancestral haplotypes HLA A-2, B44, and DR4 [15]. Pneumonia is an important cause of morbidity and mortality among HIV-infected patients. In a study of 240 HIV-infected individuals in Chile, pneumonia was the defining disease of AIDS in 50% of patients [16]. However, pneumonitis due to CMV rarely presents but when it does, it is associated with important morbidity and mortality in immunocompromised patients [12]. In Hematopoietic Cell Transplant (HCT) recipients, CMV is a major cause of morbidity and mortality [17]. In HIV-patients, there is increasing data suggesting that CMV has an indirect effect on HIV pathogenesis, leading to increased rates of transmission and progression when a patient has a coinfection and even increasing mortality by avoiding restoration of CD4+ T-cells [18]. Latency in lung alveolar macrophages and pulmonary epithelial cells has been demonstrated in human studies, although the mechanism of invasion is still unclear. CMV replication correlates with the degree of immunosuppression, where HCT patients are more frequently and severely affected compared to other immunosuppressed populations [17]. Initially we considered the possibility of* P. jirovecii* pneumonia, and this could have led to IRIS because of OI's nonoptimal treatment before ART.

There are only a few publications describing CMV pneumonia; however we identified one study suggesting to physicians suspecting this diagnosis in HIV-patients who present with bilateral infiltrates on X-ray and lack of identification of a responsible microorganism after bronchoalveolar lavage, pointing out that the identification of CMV by this method is unreliable [19]; therefore, biopsy becomes necessary, as was stated in our patient. Bilateral interstitial infiltrates are the most common finding in chest X-ray, a finding that was observed in our clinical case. Computer tomography has a higher sensitivity and can assist in the early detection of subtler pulmonary infiltrates that were ground-glass air spaces opacities or patchy infiltrates, and small centrilobular nodules are the most common findings. A diffuse interstitial process can be seen less frequently [17]. In addition to these findings, our patient also developed node involvement, showing the systemic implication of the disease. Other diagnostic tests include viral culture in human fibroblastoid cells; however, it takes up to 4 weeks for virus detection, making it impractical for prompt diagnosis. With quantitative PCR with higher sensitivity, nevertheless, the presence of CMV DNA in bronchoalveolar lavages (BAL) from asymptomatic patients has been described, which makes the interpretation of a positive result a real challenge. Cytopathology of lung biopsies has the highest specificity but low sensitivity, although it may be enhanced by immunohistochemical staining with anti-CMV antibodies. Shell vial culture or direct fluorescent antigen testing is used for rapid detection, which has proven to be highly sensitive for the detection of CMV pneumonia; the virus is identified, using monoclonal antibodies tagging immediate-early antigen within 24 hours, whereas direct fluorescent antibody has low sensitivity but is useful as a rapid test [17].

Even though cytopathology of the lung biopsy with the identification of typical intranuclear inclusions is the most specific test for CMV pneumonia, it is not always possible to perform, like in severe cases with rapid onset respiratory failure, patients with mechanical ventilation, or thrombocytopenic cases. Fine needle aspiration and less invasive pathologic sampling may not be suitable for CMV pneumonia diagnosis, since they can miss relevant clinical diseases. In our case report, the open lung biopsy allowed us to establish the diagnosis; fortunately we could perform the procedure before the patient developed a critical status.

The association of IRIS with CMV has only been stated in ocular affections due to CMV such as vitritis, uveitis, or retinitis [20]. One study described that up to 37.7% of patients with a diagnosis of CMV-retinitis prior to antiretroviral therapy (ART) initiation developed IRIS, compared to 6.4% patients with a diagnosis of KS [5]. There are currently no defined criteria for immune recovery uveitis (IRU); nevertheless it is recognized in AIDS patients with cytomegalovirus retinitis who develop new or increased noninfectious intraocular inflammatory reactions a few weeks after ART initiation [20]. Regarding treatment for CMV, published data agrees on ganciclovir IV, with cidofovir and foscarnet as alternatives. The treatment can be switched to valganciclovir PO after two weeks of intravenous treatment after demonstrating patient's clinical and radiological improvement; keeping the therapy until the maintenance of the CD4+ T-cell count is above 100 for 3–6 months [19]. Our patient, was treated with ganciclovir 500 mg IV, once the diagnosis of CMV pneumonitis was established and continued for 17 days; additionally to decrease the exacerbated inflammatory response, the patient required both anti-inflammatory drugs (methylprednisolone) and immunomodulatory drugs (thalidomide).

## 4. Conclusion

CMV pneumonia could represent a life-threatening condition specially in patients that develop IRIS; in them an aggressive anti-inflammatory and immunosuppressive treatment might be required and should be considered. The published evidence and our clinical experience suggest the importance and urgent need to develop preventive strategies as well as specific and universal diagnostic criteria and therapeutic guidelines, based on the pathogenesis of IRIS, to reduce mortality and improve quality of life in HIV-patients with OI.

## Supplementary Material

Digitalized CT-scan of the thorax following coronal sectioning, with evidence of generalized lung involvement and mediastinal adenopathy.

## Figures and Tables

**Table 1:** Proposed criteria for the classification of IRIS patients, French et al. (2004) [13].

Major criteria	Minor criteria	Exclusion criteria
(A) Atypical presentation of opportunistic infection or tumors in patient responding to ART (B) Decrease in plasma HIV RNA level > 1 log 10 copies/ml	(1) Increase in CD4+ T-cell count after ART (2) Increase in immune response specific to relevant pathogen (3) Spontaneous resolution of disease with no specific antimicrobial therapy or tumor chemotherapy while continuing ART	(1) Failure of treatment to OIs because of microbial drug resistance (2) Poor adherence to treatment for OI/absorption problems (3) Presence of other OIs or neoplasm, drug toxicity, or reaction

For IRIS diagnosis patients must meet 2 major criteria (A + B) or one major criterion (A or B) plus 2 minor criteria (1, 2, or 3), without the presence of exclusion criteria, IRIS immune reconstitution inflammatory syndrome, ART antiretroviral therapy, OIs opportunistic infections, and HIV human immunodeficiency virus.

## Abbreviations

IRIS: Immune reconstitution inflammatory syndrome
HIV: Human immunodeficiency virus
AIDS: Acquired immunodeficiency syndrome
CMV: Cytomegalovirus
ART: Antiretroviral treatment
OI: Opportunistic infections.
